# Left Atrial Appendage Occlusion Versus Medical Therapy in Atrial Fibrillation: A Systematic Review and Meta-Analysis

**DOI:** 10.3390/jcm15145529

**Published:** 2026-07-15

**Authors:** Muhammad Aslam Khan, Anza Muhammad, Sheeza Nawaz, Muhammad Khan Buhadur Ali, Muhammad Shahzaib, Aleena Sameen, Maheen Anwar, Akriti Agrawal, Hitesh Bhatia, Syed Zamrak Khan, Saurabh Sharma

**Affiliations:** 1 Department of Internal Medicine, Guthrie Clinic Robert Packer Hospital, Sayre, PA 18840, USA; ghilzai.md@gmail.com (M.A.K.); maheen.anwar@guthrie.org (M.A.); akriti.agrawal@guthrie.org (A.A.); 2 Department of Medicine, King Edward Medical University, Lahore 54000, Pakistan; anzamuhammed@gmail.com (A.M.); buhadurali56@gmail.com (M.K.B.A.); muhammadshahzaib@kemu.edu.pk (M.S.); aleena.sameen@yahoo.com (A.S.); 3 Department of Cardiology, Guthrie Clinic Robert Packer Hospital, Sayre, PA 18840, USAsaurabh.sharma@guthrie.org (S.S.); 4 Department of Cardiology, University of Cincinnati, Cincinnati, OH 45267-0542, USA

**Keywords:** left atrial appendage occlusion, atrial fibrillation, anticoagulation, meta-analysis, bleeding, stroke prevention

## Abstract

**Background:** Percutaneous left atrial appendage occlusion (LAAO) is an established nonpharmacologic strategy for stroke prevention in atrial fibrillation (AF). Its comparative effectiveness and safety relative to contemporary medical therapy, including direct oral anticoagulants (DOACs), remain uncertain following recent large randomized controlled trials (RCTs). **Methods:** We performed a systematic review and meta-analysis of RCTs comparing catheter-based LAAO with medical therapy in AF patients. PubMed, CENTRAL, and ScienceDirect were searched from inception through May 2026. Dichotomous outcomes were pooled as risk ratios (RRs) with 95% confidence intervals (CIs) using random-effects models. The primary outcome was the composite primary endpoint. Secondary outcomes included all-cause death, cardiovascular death, all stroke/TIA, ischemic stroke/TIA, systemic embolism, major bleeding, and nonprocedural major bleeding. Risk of bias was assessed using ROB 2. **Results:** Six RCTs were included, contributing 7073 participants (3729 LAAO; 3344 medical therapy). LAAO was not associated with significant differences in the composite endpoint (RR 1.02; 95% CI 0.85–1.23), all-cause death (RR 1.02; 95% CI 0.79–1.31), cardiovascular death (RR 0.93; 95% CI 0.67–1.29), all stroke/TIA (RR 1.06; 95% CI 0.81–1.38), ischemic stroke/TIA (RR 1.24; 95% CI 0.88–1.76), systemic embolism (RR 0.76; 95% CI 0.12–4.77), or total major bleeding (RR 0.93; 95% CI 0.77–1.13). LAAO significantly reduced nonprocedural bleeding (RR 0.54; 95% CI 0.46–0.63; *p* < 0.0001; I^2^ = 0.0%). Heterogeneity was low to moderate across outcomes. **Conclusions:** No significant differences were observed between LAAO and medical therapy for the composite endpoint, mortality, or thromboembolic outcomes; however, confidence intervals for ischemic stroke/TIA and systemic embolism remained wide and cannot exclude a clinically meaningful excess of thromboembolic events after LAAO. LAAO was associated with a substantial and consistent reduction in nonprocedural bleeding. These findings suggest LAAO may be considered an individualized alternative to oral anticoagulation for selected patients with high bleeding risk or anticoagulation intolerance, weighing upfront procedural risk against this bleeding benefit, while uncertainty for rare thromboembolic outcomes remains to be resolved.

## 1. Introduction

Atrial fibrillation is a major cause of preventable ischemic stroke and systemic embolism, and long-term oral anticoagulation remains the cornerstone of stroke prevention for patients with elevated thromboembolic risk [1,2,3,4]. Direct oral anticoagulants (DOACs) have improved the safety and practicality of anticoagulation compared with vitamin K antagonists, demonstrating at least comparable efficacy and lower intracranial bleeding risk relative to warfarin [5,6,7,8,9]. Nevertheless, clinically important limitations persist, including bleeding complications, treatment interruption, renal dysfunction, and adherence challenges.

Percutaneous left atrial appendage occlusion (LAAO) was developed as a nonpharmacologic strategy to reduce thromboembolic risk by excluding the dominant anatomic source of thrombus formation in nonvalvular atrial fibrillation [10,11]. Early randomized evidence established LAAO as an alternative to warfarin, and longer-term pooled data suggested durable thromboembolic protection with reductions in selected bleeding outcomes over time [12,13,14]. More recent randomized trials have compared contemporary device-based closure with DOAC-based or broader medical therapy strategies, including the CHAMPION-AF and CLOSURE-AF trials published in 2026 [15,16,17,18,19].

Despite increasing LAAO adoption, uncertainty persists regarding its comparative effectiveness relative to contemporary medical therapy. Individual trials have differed in patient risk profile, comparator regimen, device platform, follow-up duration, and endpoint definitions, making pooled assessment valuable for estimating the balance between thromboembolic prevention, mortality, and bleeding outcomes [20]. We performed a systematic review and meta-analysis of randomized evidence comparing LAAO with medical therapy in AF patients, hypothesizing that LAAO would provide comparable protection against mortality and thromboembolic outcomes while reducing nonprocedural bleeding.

## 2. Methods

### 2.1. Reporting Framework

This systematic review and meta-analysis was conducted in accordance with the PRISMA 2020 statement (Supplementary Table S1) [21]. The review protocol is accessible via the PROSPERO database (CRD420261403066). No amendments were made to the registered protocol.

### 2.2. Search Strategy

A systematic literature search was performed in PubMed, CENTRAL, and ScienceDirect from inception through May 2026. The search strategy included controlled vocabulary and free-text terms for left atrial appendage closure/occlusion and device names (Watchman, LARIAT, Amplatzer, LAmbre), atrial fibrillation terms, and comparator terms including anticoagulation, warfarin, dabigatran, apixaban, rivaroxaban, DOAC, antiplatelet therapy, and aspirin. The full search strategy is provided in the Supplementary Table S2.

### 2.3. Eligibility Criteria

Eligible studies were randomized controlled trials enrolling patients with AF and comparing catheter-based LAAO with medical therapy. Studies were required to report at least one prespecified clinical outcome. Exclusion criteria included observational studies, single-arm studies, non-AF populations, surgical LAA exclusion, conference-only abstracts, and duplicate reports.

### 2.4. Study Selection and Data Extraction

Titles, abstracts, and full texts were screened independently by two reviewers (AM and AS), with disagreements resolved by consensus. Extracted variables included study design, intervention and comparator, sample size, sex, age, body mass index, comorbidities, CHA2DS2-VASc score, AF pattern, outcome definitions, event counts, and denominators.

### 2.5. Outcomes

The primary outcome was the composite primary endpoint as defined by each trial. Secondary outcomes included all-cause death, cardiovascular death, all stroke/TIA, ischemic stroke/TIA, systemic embolism, major bleeding, major bleeding not related to the device/procedure, and procedure- or device-related complications.

### 2.6. Risk of Bias Assessment

Risk of bias was assessed using the Cochrane Risk of Bias 2 (ROB 2) tool for individually randomized, parallel-group trials [22,23]. Each domain and the overall risk of bias were rated as low risk, some concerns, or high risk.

### 2.7. Statistical Analysis

**Figure A1:**
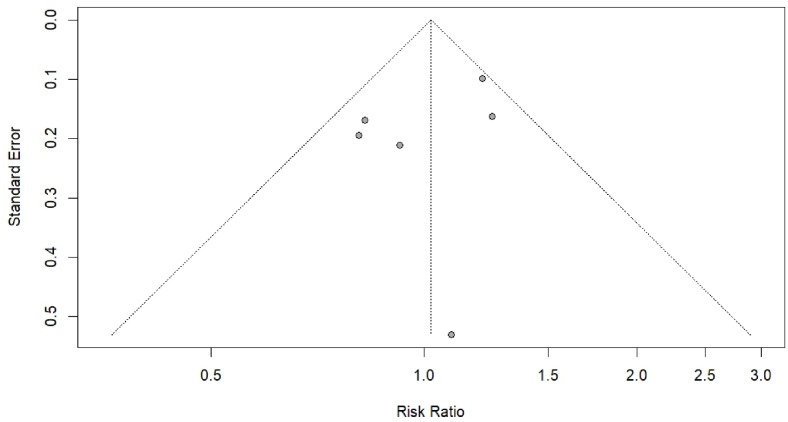
Funnel plot for the composite primary endpoint.

**Figure A2:**
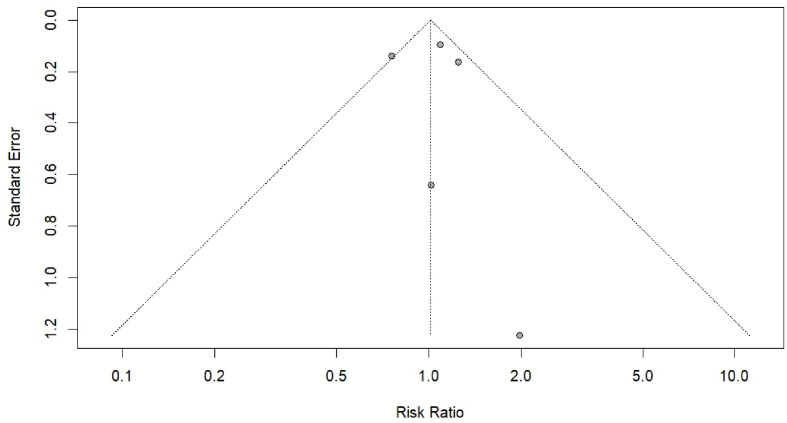
Funnel plot for all-cause death.

**Figure A3:**
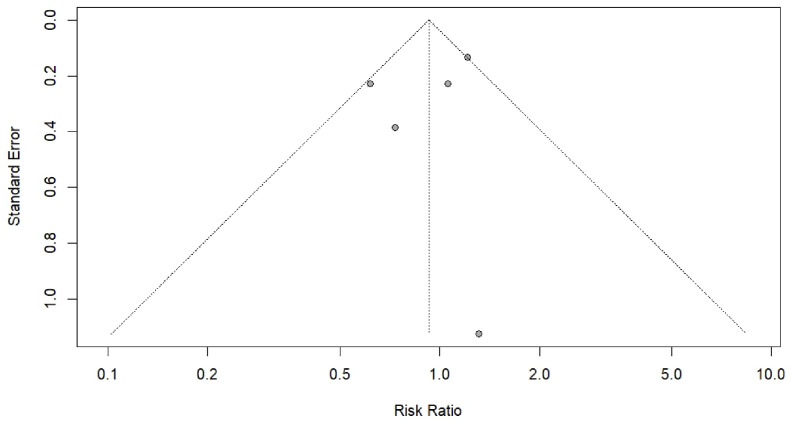
Funnel plot for cardiovascular death.

**Figure A4:**
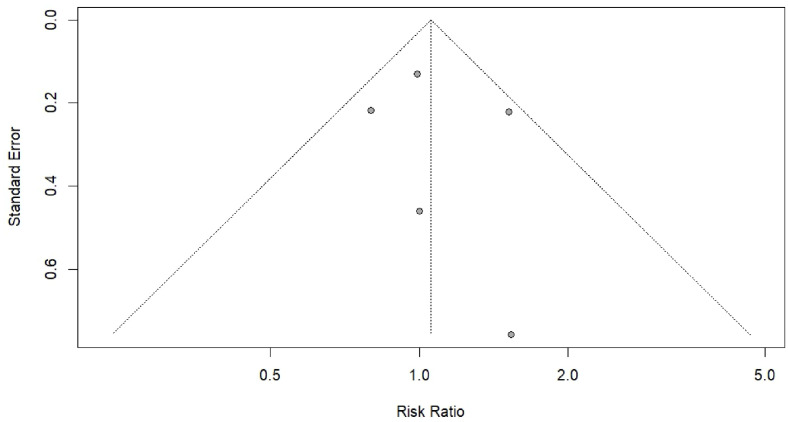
Funnel plot for all stroke/TIA.

**Figure A5:**
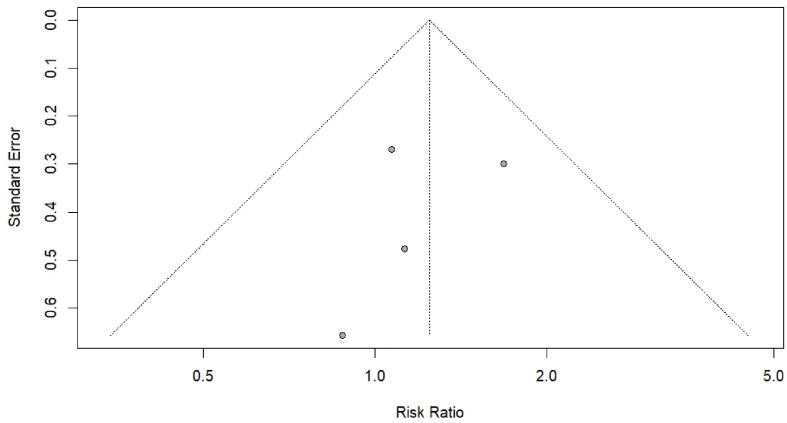
Funnel plot for ischemic stroke/TIA.

**Figure A6:**
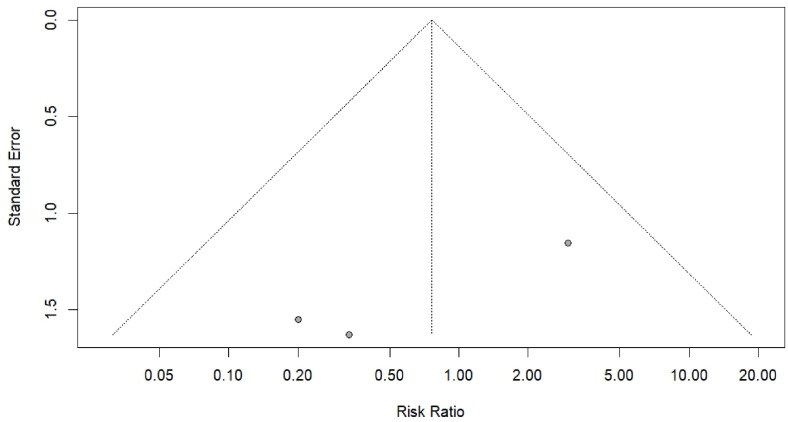
Funnel plot for systemic embolism.

**Figure A7:**
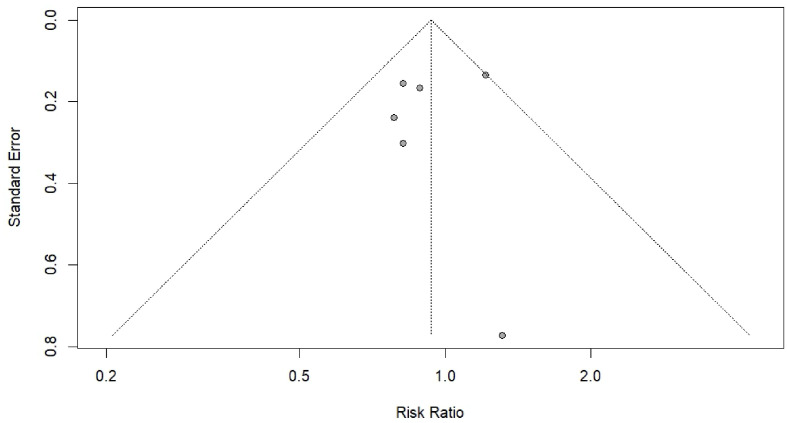
Funnel plot for major bleeding.

**Figure A8:**
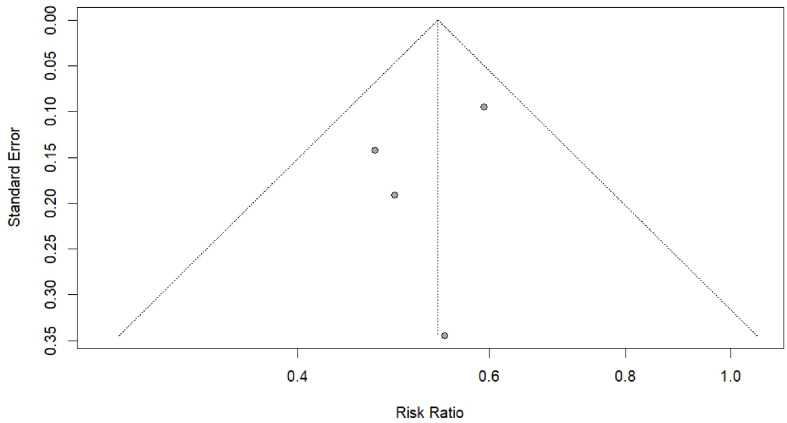
Funnel plot for major bleeding not related to the device/procedure.

**Figure A9:**
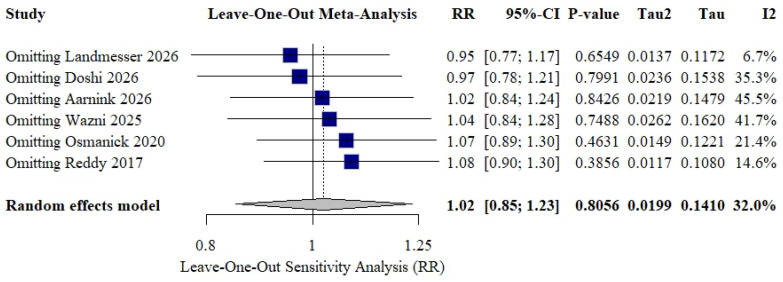
Leave-one-out sensitivity analysis for the composite primary endpoint [14,15,16,17,18,19].

**Figure A10:**
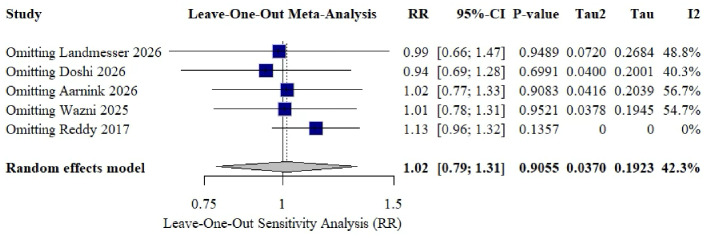
Leave-one-out sensitivity analysis for all-cause death [14,16,17,18,19].

**Figure A11:**
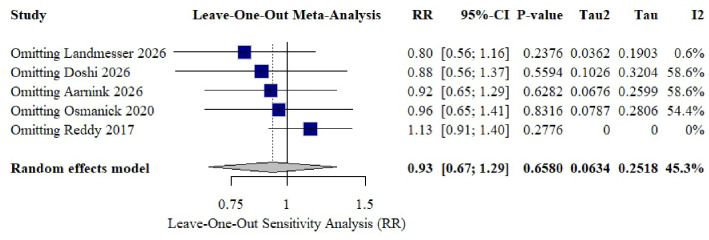
Leave-one-out sensitivity analysis for cardiovascular death [14,15,17,18,19].

**Figure A12:**
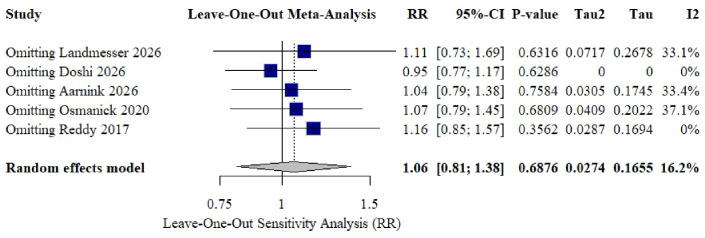
Leave-one-out sensitivity analysis for all stroke/TIA [14,15,17,18,19].

**Figure A13:**
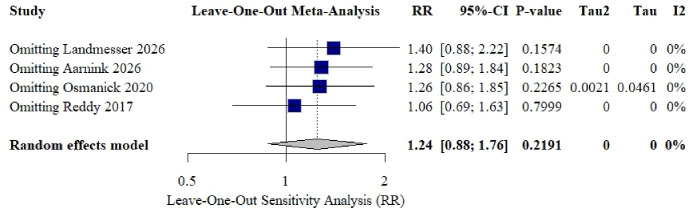
Leave-one-out sensitivity analysis for ischemic stroke/TIA [14,15,18,19].

**Figure A14:**
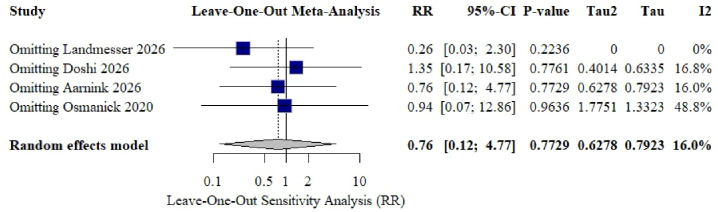
Leave-one-out sensitivity analysis for systemic embolism [15,17,18,19].

**Figure A15:**
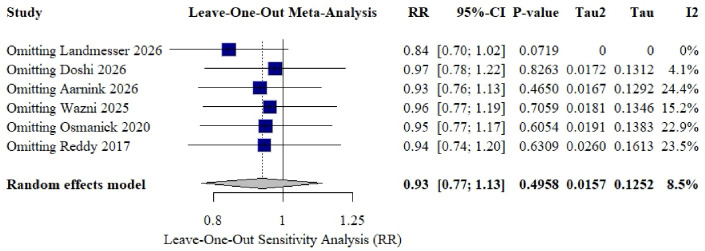
Leave-one-out sensitivity analysis for major bleeding [14,15,16,17,18,19].

**Figure A16:**
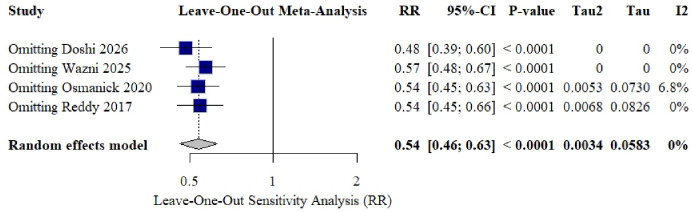
Leave-one-out sensitivity analysis for major bleeding not related to the device/procedure [14,15,16,17].

**Figure A17:**
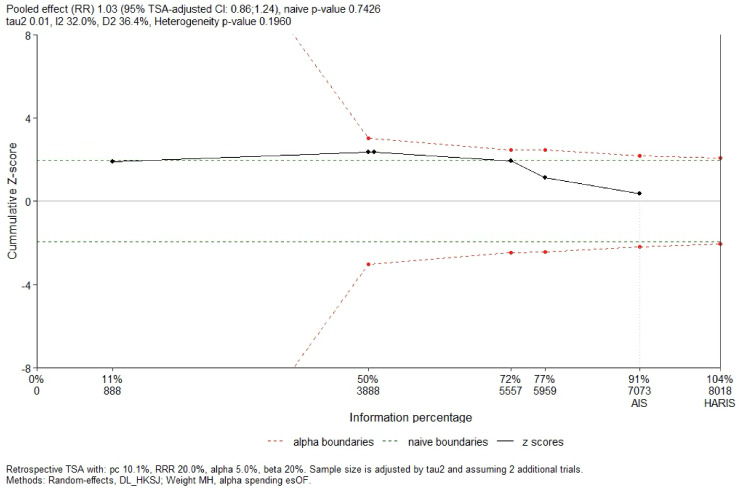
Trial sequential analysis for the composite primary endpoint.

**Figure A18:**
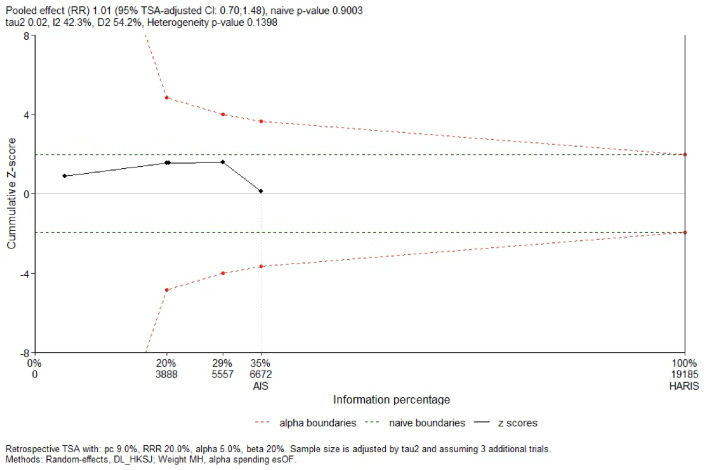
Trial sequential analysis for all-cause death.

**Figure A19:**
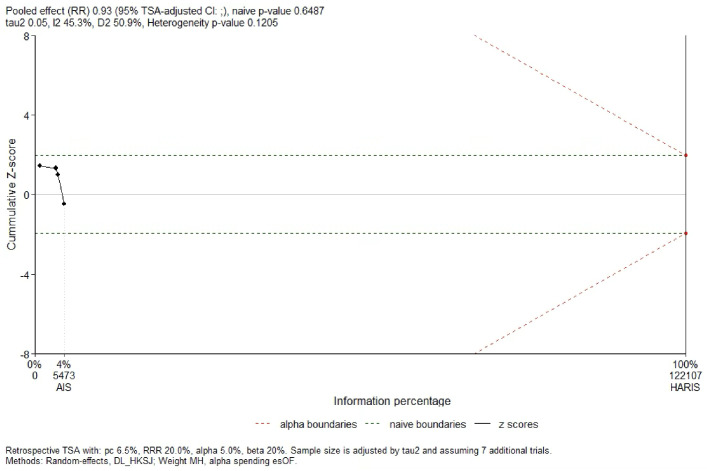
Trial sequential analysis for cardiovascular death.

**Figure A20:**
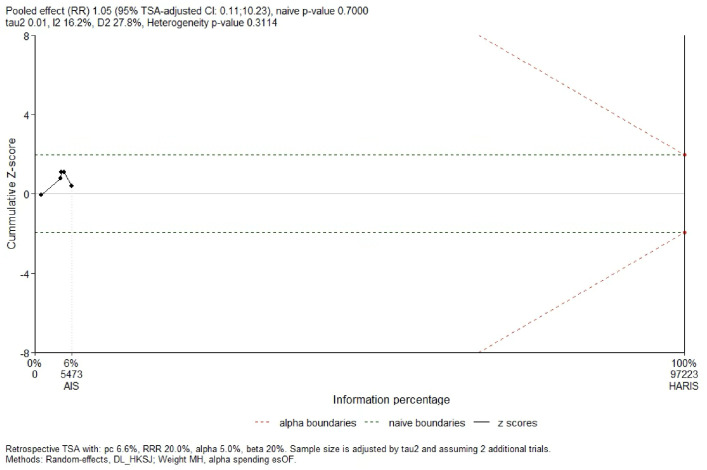
Trial sequential analysis for all stroke/TIA.

**Figure A21:**
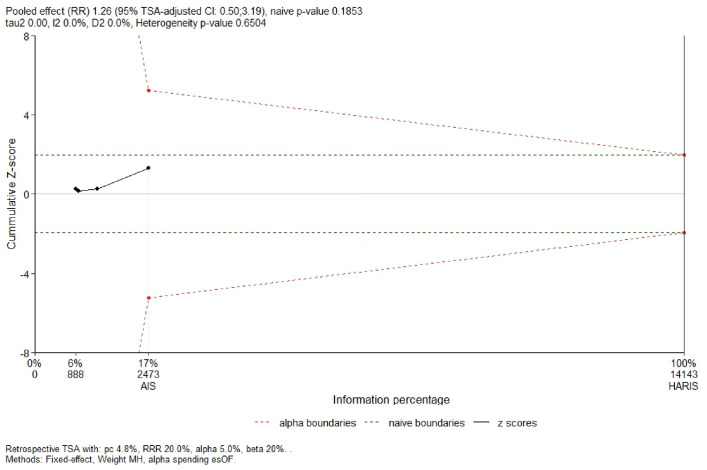
Trial sequential analysis for ischemic stroke/TIA.

**Figure A22:**
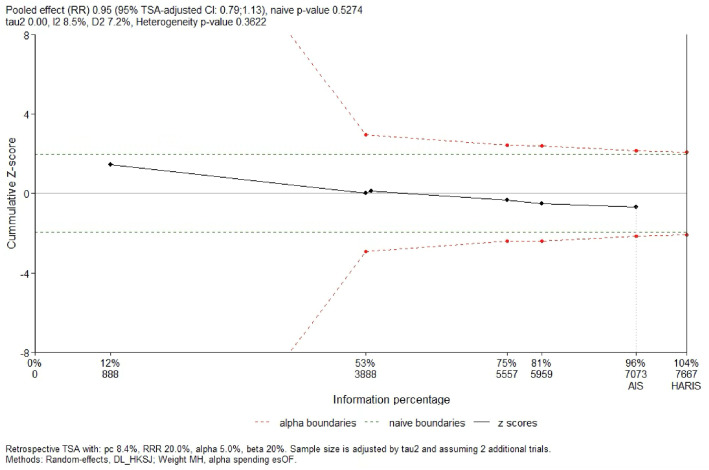
Trial sequential analysis for major bleeding.

**Figure A23:**
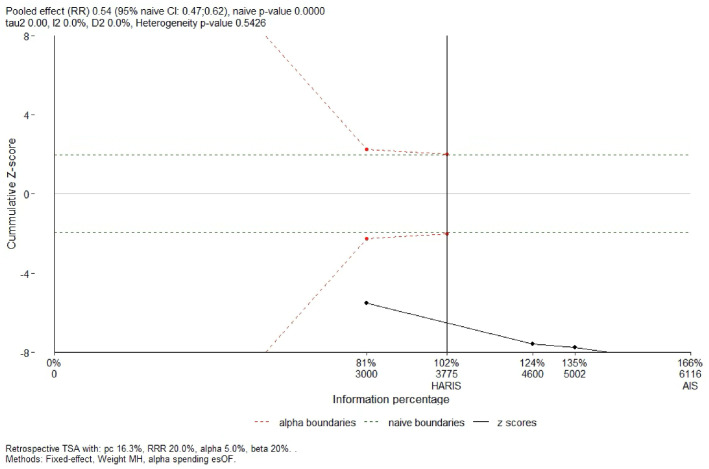
Trial sequential analysis for major bleeding not related to the device/procedure.

Dichotomous outcomes were pooled as risk ratios (RRs) with 95% CIs using random-effects models (DerSimonian–Laird method) [24]. Between-study heterogeneity was assessed using I^2^, τ^2^, and the Cochran Q test [25]. Overall effects were evaluated using z-tests. Leave-one-out sensitivity analyses were performed for each outcome [Figure A9, Figure A10, Figure A11, Figure A12, Figure A13, Figure A14, Figure A15 and Figure A16]. Funnel plots were generated to assess for potential publication bias [26] [Figure A1, Figure A2, Figure A3, Figure A4, Figure A5, Figure A6, Figure A7 and Figure A8]. Trial sequential analysis was performed for selected outcomes [27] [Figure A17, Figure A18, Figure A19, Figure A20, Figure A21, Figure A22 and Figure A23]. Analyses were performed in R (version 4.5.0) using packages meta and rtsa [28,29]. Certainty of evidence for the eight main clinical outcomes was assessed using the GRADE approach, considering risk of bias, inconsistency, indirectness, imprecision, and publication bias [30].

## 3. Results

### 3.1. Study Selection

A total of 1809 records were identified through database searches; 596 duplicate records were removed before screening. Six randomized study records were included in the qualitative and quantitative synthesis, contributing 7073 participants (3729 LAAO; 3344 medical therapy). The included studies were Landmesser 2026, Doshi 2026, Aarnink 2026, Wazni 2025, Osmancik 2020, and Reddy 2017 [14,15,16,17,18,19] [Supplementary Figure S1]. Studies excluded after full-text review were primarily non-randomized in design, reported duplicate data from included trials, or did not report any prespecified clinical outcome; a full list of excluded studies with reasons is available from the corresponding author upon request.

### 3.2. Study Characteristics

All included studies were randomized controlled comparisons of catheter-based LAAO versus medical therapy (Table 1). Landmesser 2026 (CLOSURE-AF) compared LAAO with physician-directed best medical care including DOACs if eligible [18]. Doshi 2026 (CHAMPION-AF) compared device-based LAAO with NOAC therapy [17]. Aarnink 2026 (COMPARE-LAAO) compared LAAO with antiplatelet therapy or no antithrombotic therapy in patients ineligible for anticoagulation [19]. Wazni 2025 (OPTION) compared LAAO after catheter ablation with oral anticoagulation [16]. Osmancik 2020 (PRAGUE-17) compared LAAO with DOAC therapy [15]. Reddy 2017 reported pooled PREVAIL/PROTECT AF data comparing Watchman closure with warfarin [14]. Trial sizes ranged from 69 participants (Aarnink 2026) [19] to 3000 (Doshi 2026) [17]. Mean age ranged from 69.4 years (Wazni 2025) [16] to 78.5 years (Landmesser 2026 LAAO arm) [18]. Baseline CHA2DS2-VASc scores ranged from 3.5 to 5.2. Baseline demographics and comorbidities are presented in Table 2A,B.

### 3.3. Risk of Bias

Landmesser 2026 [18] and Doshi 2026 [17] were judged overall low risk of bias. Aarnink 2026 [19], Wazni 2025 [16], and Osmancik 2020 [15] were judged as having some concerns, primarily in deviations from intended interventions and outcome measurement. Reddy 2017 [14] was judged as having some concerns across multiple domains including randomization and selection of reported results [Supplementary Figure S2].

### 3.4. Primary Outcome

For the composite primary endpoint, six study records contributed 3729 LAAO and 3344 medical-therapy participants. LAAO was not associated with a significant difference in the composite endpoint (RR 1.02; 95% CI 0.85–1.23; *p* = 0.8056; I^2^ = 32.0%; τ^2^ = 0.0199; Supplementary Figure S3).

Pooled estimates for all outcomes are presented in Table 3.

### 3.5. Mortality Outcomes

All-cause death (5 studies; 3528 LAAO; 3143 medical therapy) was not significantly different between groups (RR 1.02; 95% CI 0.79–1.31; *p* = 0.9055; I^2^ = 42.3%; Supplementary Figure S4). Cardiovascular death (5 studies; 2926 LAAO; 2547 medical therapy) was also similar between strategies (RR 0.93; 95% CI 0.67–1.29; *p* = 0.6580; I^2^ = 45.3%; Supplementary Figure S5).

### 3.6. Thromboembolic Outcomes

All stroke/TIA (5 studies; 2926 LAAO; 2547 medical therapy) did not differ significantly between strategies (RR 1.06; 95% CI 0.81–1.38; *p* = 0.6876; I^2^ = 16.2%; Supplementary Figure S6). Ischemic stroke/TIA (4 studies; 1427 LAAO; 1046 medical therapy) showed no significant difference (RR 1.24; 95% CI 0.88–1.76; *p* = 0.2191; I^2^ = 0.0%; Supplementary Figure S7). Systemic embolism was rare and the pooled estimate was imprecise (4 studies; RR 0.76; 95% CI 0.12–4.77; *p* = 0.7729; I^2^ = 16.0%; Supplementary Figure S8).

### 3.7. Bleeding and Procedural Outcomes

**Figure 1:**
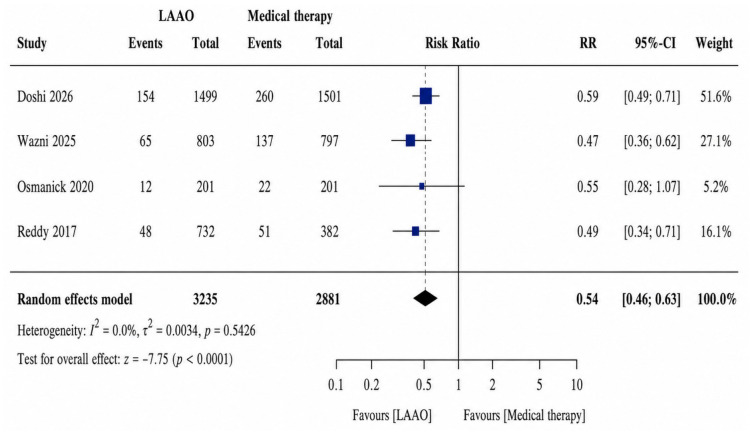
Forest plot for major bleeding not related to the device/procedure [14,15,16,17].

Total major bleeding did not differ significantly between groups (6 studies; 3729 LAAO; 3344 medical therapy; RR 0.93; 95% CI 0.77–1.13; *p* = 0.4958; I^2^ = 8.5%; Supplementary Figure S9). In contrast, LAAO was associated with a marked and statistically significant reduction in major bleeding not related to the device or procedure (4 studies; 3235 LAAO; 2881 medical therapy; RR 0.54; 95% CI 0.46–0.63; *p* < 0.0001; I^2^ = 0.0%; Figure 1). Procedure and device related complications were reported only for LAAO arms and were not directly comparable across trials owing to differing definitions and reporting windows; descriptive trial-level rates for peri-procedural stroke/TIA, pericardial tamponade, vascular complications, device embolization, device-related thrombus, peri-device leak, procedure-related bleeding, and implantation success are presented in Table 4, including data from the original PROTECT AF and PREVAIL trial publications underlying the pooled Reddy 2017 analysis [12,13,14,15,16,17,18,19]. A pooled comparative estimate across arms was not calculable.

### 3.8. Sensitivity Analyses

Leave-one-out analyses did not materially alter the direction or statistical interpretation of any neutral outcome [Figure A8, Figure A9, Figure A10, Figure A11, Figure A12, Figure A13, Figure A14, Figure A15 and Figure A16]. RR ranges were: all stroke/TIA (0.95–1.16), ischemic stroke/TIA (1.06–1.40), systemic embolism (0.26–1.35), all-cause death (0.94–1.13), cardiovascular death (0.80–1.13), major bleeding (0.84–0.97, all CIs crossing 1.0), and composite endpoint (0.95–1.08). The nonprocedural bleeding reduction remained significant in every leave-one-out iteration (RR range 0.48–0.57; *p* < 0.0001 throughout).

### 3.9. Publication Bias

Funnel plots were generated for all eight outcomes [Figure A1, Figure A2, Figure A3, Figure A4, Figure A5, Figure A6, Figure A7 and Figure A8]. Given that each outcome included six or fewer studies, formal quantitative testing for asymmetry was limited in interpretive value [26].

### 3.10. Certainty of Evidence

Certainty of evidence for the eight main clinical outcomes was rated using the GRADE approach [30] (Table 5). All outcomes were downgraded for indirectness because included trials compared LAAO against heterogeneous medical-therapy regimens (warfarin, direct oral anticoagulants, antiplatelet therapy, or no antithrombotic therapy) in populations that differed in eligibility for anticoagulation and baseline thromboembolic/bleeding risk, and for risk of bias given that four of six trials were judged to have some concerns or high risk of bias on ROB 2 and that an open-label, unblinded design is inherent to all LAAO-versus-medical-therapy comparisons. Certainty was further downgraded for inconsistency where between-study heterogeneity was moderate (all-cause death, cardiovascular death) and for imprecision where confidence intervals were wide relative to the event rate (cardiovascular death, ischemic stroke/TIA, systemic embolism). Certainty was not downgraded for publication bias, as formal testing was judged to have limited interpretive value given six or fewer studies per outcome. Overall, certainty ranged from low for the composite endpoint, all stroke/TIA, major bleeding, and nonprocedural major bleeding, to very low for all-cause death, cardiovascular death, ischemic stroke/TIA, and systemic embolism, reflecting genuine residual uncertainty for thromboembolic outcomes that should temper any inference of comparative equivalence.

## 4. Discussion

This meta-analysis of six randomized trials enrolling 7073 participants found no statistically significant differences between catheter-based LAAO and medical therapy for all-cause death, cardiovascular death, all stroke/TIA, ischemic stroke/TIA, systemic embolism, or the composite primary endpoint; the absence of a significant difference should not be equated with proven equivalence, particularly for ischemic stroke/TIA, where the point estimate numerically favored medical therapy. The most consistent and clinically significant finding was a 46% relative reduction in nonprocedural bleeding with LAAO (RR 0.54; 95% CI 0.46–0.63; *p* < 0.0001; I^2^ = 0.0%; Figure 1). Statistical heterogeneity was low to moderate across most outcomes, which supports the consistency of these findings despite variations in trial design, patient risk profiles, comparator therapies, and device generations. Contemporary guidelines and expert statements continue to frame LAAO as a patient-selected alternative rather than a universal replacement for oral anticoagulation [31,32]. For ischemic stroke/TIA, the upper confidence bound (RR 1.76) remains compatible with a clinically relevant excess of ischemic events after LAAO, and this residual uncertainty, rather than equivalence, is the more accurate characterization of the current evidence (Table 5).

LAAO did not improve mortality or ischemic/thromboembolic outcomes compared with medical therapy. No significant differences were observed in all-cause mortality (RR 1.02; 95% CI 0.79–1.31; *p* = 0.9055; I^2^ = 42.3%), cardiovascular death (RR 0.93; 95% CI 0.67–1.29; *p* = 0.6580; I^2^ = 45.3%), all stroke/TIA (RR 1.06; 95% CI 0.81–1.38; *p* = 0.6876; I^2^ = 16.2%), ischemic stroke/TIA (RR 1.24; 95% CI 0.88–1.76; *p* = 0.2191; I^2^ = 0.0%), or systemic embolism (RR 0.76; 95% CI 0.12–4.77; *p* = 0.7729; I^2^ = 16.0%). These findings align with the current trial landscape. CHAMPION-AF demonstrated non-inferiority of LAAO versus DOACs for cardiovascular death, stroke, or systemic embolism, whereas CLOSURE-AF did not establish non-inferiority for a broader composite endpoint in a higher-risk population [17,18]. The five-year pooled PREVAIL/PROTECT AF analysis showed comparable long-term efficacy versus warfarin [14]. A network meta-analysis similarly found no significant difference in stroke or systemic embolism between LAAO and either vitamin K antagonists or DOACs [20]. Collectively, these data support LAAO as an alternative rather than a superior strategy for thromboembolic prevention. However, residual uncertainty, particularly for rare events such as ischemic stroke/TIA and systemic embolism, precludes a firm conclusion of equivalent thromboembolic protection, and certainty of evidence for these outcomes was rated very low (Table 5).

The moderate heterogeneity observed for all-cause mortality (I^2^ = 42.3%) and cardiovascular death (I^2^ = 45.3%) warrants further discussion, whereas heterogeneity was lower for all stroke/TIA (I^2^ = 16.2%) and absent for ischemic stroke/TIA (I^2^ = 0.0%). In earlier warfarin-era trials, LAAO appeared to offer a directional advantage for stroke prevention, whereas CHAMPION-AF reported a numerically higher stroke rate in the device group, partly due to peri-procedural events [12,13,14,17]. For cardiovascular death, PROTECT AF indicated a potential benefit for LAAO that was not consistently reproduced in more recent DOAC-era comparisons [14,17,18]. These trends likely reflect differences in comparator therapies, baseline patient risk, and procedural eras. Systemic embolism was rare, and the pooled estimate was imprecise (RR 0.76; 95% CI 0.12–4.77). Previous randomized trial meta-analyses similarly emphasize that estimates for infrequent thromboembolic endpoints remain sensitive to event scarcity and trial design [31].

The most consistent and clinically significant finding was the reduction in nonprocedural bleeding (RR 0.54; 95% CI 0.46–0.63; *p* < 0.0001; I^2^ = 0.0%). This substantial effect was observed without heterogeneity and is corroborated by CHAMPION-AF, extended follow-up from PRAGUE-17, and the OPTION trial, which reported lower non-procedure-related bleeding with LAAO following catheter ablation [16,17,33]. The consistency of this finding across devices and anticoagulation comparators indicates that avoidance of chronic anticoagulant exposure is the principal clinical benefit of LAAO.

When procedural and nonprocedural major bleeding events were combined, no significant difference was observed (RR 0.93; 95% CI 0.77–1.13). This finding highlights the temporal nature of the bleeding tradeoff: LAAO reduces chronic pharmacologic bleeding risk but introduces an initial procedural hazard. Investigators from CLOSURE-AF noted that high-risk patients are especially vulnerable to peri-procedural complications, and longer-term PRAGUE-17 data suggest that the bleeding benefit of LAAO emerges from reductions in late events [18,32]. Thus, the clinical advantage of LAAO may accumulate over time. The neutral pooled efficacy-and-safety composite (RR 1.02; 95% CI 0.85–1.23) also reflects these offsetting effects. Net clinical benefit frameworks may provide a more informative assessment in this context, particularly when bleeding risk is a key factor in treatment selection [34].

Several contextual factors warrant consideration. Patient populations differed across trials: CLOSURE-AF enrolled an older, higher-risk cohort; CHAMPION-AF included patients suitable for long-term DOAC therapy; PRAGUE-17 represented a high-risk population with increased thromboembolic and bleeding risk; OPTION focused on a post-ablation population; and COMPARE-LAAO enrolled patients ineligible for oral anticoagulation [15,16,17,18,19]. Comparator therapy evolved from warfarin to DOACs over the trial era, reducing the expected relative advantage of LAAO with respect to intracranial bleeding. Surgical LAAO data, including LAAOS III, further support the biological relevance of appendage exclusion, but those results cannot be directly extrapolated to stand-alone percutaneous LAAO because surgical closure was tested as an adjunct to cardiac surgery with background anticoagulation [35]. Device technology also evolved over time, and head-to-head device studies and device-related thrombus analyses underscore that procedural safety, occlusion completeness, post-procedure anti-thrombotic regimens, and device platform may influence outcomes [33,36]. Beyond device generation, implantation technique itself appears to modulate device-related outcomes: in a dual-center cohort of 236 patients undergoing Watchman FLX implantation, controlled device over-compression (>30%) was independently associated with significantly lower residual peri-device leak at 2-month trans-esophageal echocardiographic follow-up (8.2% versus 32.6%; *p* < 0.001) without an increase in procedural complications, suggesting that operator technique, independent of device platform, may meaningfully influence long-term sealing efficacy [37].

Three specific subgroups warrant additional comment because trial-level pooling cannot resolve potential effect modification within them. With respect to advanced age, several included trials performed pre-specified subgroup analyses (typically dichotomized at 75 years) without a significant treatment-by-age interaction [12,14,15]; however, CLOSURE-AF specifically enrolled an older, higher-risk cohort (mean age 77.9–78.5 years) and did not achieve non-inferiority [18], suggesting that age and associated frailty may modify the risk-benefit balance even when individual subgroup tests are underpowered to detect this. Consistent with this concern, a national administrative-database analysis of 84,140 LAAO recipients found that octogenarians and nonagenarians had similar adjusted odds of in-hospital mortality, stroke, and most procedural complications compared with younger patients, but significantly higher adjusted odds of vascular complications (adjusted odds ratio 1.47 for octogenarians and 1.60 for nonagenarians) [38]. Advanced age therefore appears to selectively increase access-site morbidity rather than catastrophic risk, an effect that the present trial-level meta-analysis is not powered to detect.

Diabetes mellitus is embedded within the CHA2DS2-VASc score used for trial eligibility but was not reported as an independent subgroup variable in any of the six included trials, precluding direct assessment within this meta-analysis. Observational data are reassuring on this point: a nationwide analysis of 62,220 LAAO recipients (34.9% with diabetes) found no significant difference in a composite of in-hospital death, myocardial infarction, cardiac arrest, stroke, pericardial effusion or tamponade, and post-procedural hemorrhage between patients with and without diabetes, although diabetic patients had a higher risk of acute kidney injury (3.75% versus 1.96%; *p* < 0.001) [39]. A smaller single-center series similarly reported comparable peri-procedural and 6-month outcomes between 31 diabetic and 47 non-diabetic patients despite diabetic patients carrying significantly higher baseline thromboembolic and bleeding risk scores (CHA2DS2-VASc 4.5 versus 3.5; HAS-BLED 4.7 versus 4.1; *p* < 0.001 for both) [40]. Dedicated subgroup or individual-patient-data analyses incorporating diabetes status are nonetheless warranted, given its established association with endothelial dysfunction, increased platelet activity, and impaired vascular healing, any of which could plausibly modify procedural risk even where aggregate registry data have not detected a signal.

Valvular heart disease represents a more fundamental issue than a simple unanalyzed subgroup. All six included trials enrolled patients with non-valvular AF, and where exclusion criteria were explicitly reported, mechanical valve prostheses, mitral stenosis, or significant valve disease were specifically excluded [14,15,19]. This reflects the mechanistic rationale for LAAO: the left atrial appendage is the dominant source of thrombus in nonvalvular AF, whereas in valvular, particularly rheumatic, AF, thrombi more commonly arise from the left atrial body or mitral valve apparatus rather than the appendage. The findings of this meta-analysis, therefore, cannot be extrapolated to AF associated with significant valvular heart disease, and LAAO is not mechanistically indicated in that population; such patients should continue to be managed according to guideline-directed anticoagulation or valve-specific intervention rather than LAAO.

### Limitations

First, the use of trial-level data prevented subgroup analyses by age, prior bleeding, renal dysfunction, or baseline risk scores. Second, the limited number of trials and infrequent events reduced the precision of estimates for endpoints such as systemic embolism. Third, pooling binary event counts across studies with different follow-up durations does not fully account for time-dependent risk accumulation, especially for bleeding outcomes where early procedural risk may be offset by later reductions in anticoagulant-associated bleeding. Hazard-ratio-based pooling, while in principle better suited to differential follow-up and censoring, was not adopted as the primary analysis because hazard ratios were not uniformly reported across the six included trials and the proportional-hazards assumption underlying Cox modeling was not uniformly tenable across this evidence base: CLOSURE-AF’s investigators explicitly abandoned a planned Cox model in favor of restricted mean survival time after Schoenfeld residuals indicated a violation of the proportional-hazards assumption, particularly for major bleeding [18]; PRAGUE-17 reported sub-distribution hazard ratios from a Fine-Gray competing-risk model rather than conventional cause-specific hazard ratios [15]; and COMPARE-LAAO, terminated early owing to slow enrollment, reported only descriptive annualized event rates without formal hypothesis testing [19]. Risk-ratio-based pooling of trial-level event counts was therefore retained as the primary analysis. Fourth, although risk of bias was assessed for included trials, the small number of studies limited the ability to formally assess publication bias. Fifth, heterogeneity in patient selection, endpoint definitions, comparator regimens, and device platforms restricts generalizability. Finally, a median follow-up of approximately three years may be insufficient to capture the full long-term benefit of a one-time device strategy compared with lifelong anticoagulation, although recent longer-term randomized-trial syntheses continue to support a broadly similar efficacy profile with differential bleeding tradeoffs [41].

## 5. Conclusions

LAAO should not be considered universally superior, or equivalent, to oral anticoagulation. Current randomized evidence shows no statistically significant difference between LAAO and medical therapy for thromboembolic and mortality outcomes, but confidence intervals for ischemic stroke/TIA and systemic embolism remain compatible with a clinically meaningful excess risk after LAAO, and certainty of evidence for these outcomes was rated very low; this uncertainty should not be interpreted as evidence of equivalence. LAAO substantially and consistently reduces nonprocedural bleeding, though it introduces an early procedural risk that partly offsets this benefit when procedural and nonprocedural bleeding are considered together. The greatest benefit is likely in patients for whom long-term anticoagulation is undesirable because of bleeding risk, treatment intolerance, or adherence challenges [34,42,43]. The choice between LAAO and medical therapy should therefore be individualized, weighing upfront procedural risk and residual uncertainty in thromboembolic protection against long-term bleeding exposure. Several specific avenues for future research follow directly from the gaps identified in this analysis: (1) a head-to-head randomized comparison of contemporary LAAO devices against current-generation direct oral anticoagulants specifically, rather than warfarin or heterogeneous medical-therapy regimens, to directly resolve comparative effectiveness in the DOAC era; (2) dedicated prespecified subgroup analyses, or an individual-patient-data meta-analysis, stratified by age, diabetes mellitus, and renal function, to clarify whether these characteristics modify the procedural-risk-to-bleeding-benefit balance; (3) longer-term follow-up beyond the current median of approximately three years to capture the full trajectory of the bleeding-reduction benefit relative to the upfront procedural hazard; and (4) standardized, consistently defined reporting of procedural and device-related complications across future LAAO trials to enable meaningful pooled safety estimates of the kind the present meta-analysis could not produce. Extended follow-up from contemporary randomized trials, larger pooled samples, and dedicated long-term thromboembolic safety data are needed to resolve this uncertainty and further refine patient selection.

## Figures and Tables

**Table 1:** Included randomized studies and treatment comparisons.

Study	Trial/Source	Intervention Group	Comparator Group
Landmesser 2026 [18]	CLOSURE-AF	Catheter-based LAAO	Physician-directed best medical care (DOACs if eligible)
Doshi 2026 [17]	CHAMPION-AF	Device-based LAAO	NOAC therapy
Aarnink 2026 [19]	COMPARE-LAAO	LAAO	Antiplatelet therapy or no antithrombotic therapy
Wazni 2025 [16]	OPTION	LAAO after catheter ablation	Oral anticoagulation
Osmancik 2020 [15]	PRAGUE-17	LAAO	DOAC therapy
Reddy 2017 [14]	PREVAIL/PROTECT AF pooled	Watchman LAAO	Warfarin therapy

**Table 2:** Baseline characteristics of included studies.

A. Demographic and risk-score characteristics.							
**Study**	**Arm**	**N**	**Male/Female**	**Age, Mean ± SD**	**BMI, Mean ± SD**	**CHA2DS2-VASc**	**AF Pattern**
Landmesser 2026 [18]	LAAO	446	274M/172F	78.5 ± 6.8	N/A	5.2 ± 1.5	N/A
Landmesser 2026 [18]	Med Rx	442	271M/171F	77.3 ± 7.3	N/A	5.1 ± 1.6	N/A
Doshi 2026 [17]	LAAO	1499	1014M/485F	71.6 ± 7.5	N/A	3.5 ± 1.2	Parox 1038; Persist 358; Perm 102
Doshi 2026 [17]	Med Rx	1501	1027M/473F	71.8 ± 7.5	N/A	3.5 ± 1.3	Parox 1028; Persist 382; Perm 91
Aarnink 2026 [19]	LAAO	48	35M/13F	74.5 ± 6.2	25.9 ± 6.1	4	Parox 30; Persist 2; Perm 13
Aarnink 2026 [19]	Med Rx	21	14M/7F	75.9 ± 5.7	25.5 ± 10.4	4	Parox 11; Persist 3; Perm 7
Wazni 2025 [16]	LAAO	803	520M/283F	69.4 ± 7.4	N/A	3.5 ± 1.3	Parox 477; Persist 326
Wazni 2025 [16]	Med Rx	797	533M/263F	69.4 ± 7.9	N/A	3.5 ± 1.3	Parox 296; Persist 501
Osmancik 2020 [15]	LAAO	201	134M/67F	73.2 ± 7.2	N/A	4.7 ± 1.5	Parox 53; Persist 47; LSP 18; Perm 83
Osmancik 2020 [15]	Med Rx	201	130M/71F	73.4 ± 6.7	N/A	4.7 ± 1.5	Parox 67; Persist 46; LSP 16; Perm 72
Reddy 2017 [14]	LAAO	732	508M/224F	72.6 ± 8.4	N/A	3.6 ± 1.4	Parox 330; Persist 182; Perm 202
Reddy 2017 [14]	Med Rx	382	273M/109F	73.5 ± 8.6	N/A	3.9 ± 1.5	Parox 169; Persist 89; Perm 114
B. Baseline comorbidities and prior stroke/TIA.							
**Study**	**Arm**	**Hypertension**		**Diabetes Mellitus**	**Heart Failure**	**Age > 75 y**	**Prior Stroke/TIA**
Landmesser 2026 [18]	LAAO	417 (93.5%)		175 (39.2%)	N/A	N/A	142 (31.8%)
Landmesser 2026 [18]	Med Rx	417 (94.3%)		186 (42.1%)	N/A	N/A	151 (34.2%)
Doshi 2026 [17]	LAAO	N/A		N/A	N/A	N/A	123 (8.2%)
Doshi 2026 [17]	Med Rx	N/A		N/A	N/A	N/A	124 (8.3%)
Aarnink 2026 [19]	LAAO	38 (79.2%)		8 (16.7%)	6 (12.5%)	N/A	19 (39.6%)
Aarnink 2026 [19	Med Rx	17 (81.0%)		4 (19.0%)	5 (23.8%)	N/A	9 (42.9%)
Wazni 2025 [16]	LAAO	N/A		N/A	N/A	N/A	N/A
Wazni 2025 [16]	Med Rx	N/A		N/A	N/A	N/A	N/A
Osmancik 2020 [15]	LAAO	186 (92.5%)		73 (36.3%)	88 (43.8%)	85 (42.3%)	73 (36.3%)
Osmancik 2020 [15]	Med Rx	186 (92.5%)		90 (44.8%)	90 (44.8%)	79 (39.3%)	69 (34.3%)
Reddy 2017 [14]	LAAO	652 (89.1%)		204 (27.9%)	508 (69.4%)	295 (40.3%)	161 (22.0%)
Reddy 2017 [14]	Med Rx	354 (92.7%)		113 (29.6%)	98 (25.7%)	165 (43.2%)	90 (23.6%)

Values are n (%) unless otherwise specified. N/A, data not available; Parox, paroxysmal; Persist, persistent; LSP, long-standing persistent; Perm, permanent; Med Rx, medical therapy.

**Table 3:** Pooled outcome estimates from random-effects meta-analysis.

Outcome	Study Records	Participants LAAO/Medical	RR	95% CI	*p* Value	I^2^
Composite primary endpoint	6	3729/3344	1.02	0.85–1.23	0.8056	32.0%
All-cause death	5	3528/3143	1.02	0.79–1.31	0.9055	42.3%
Cardiovascular death	5	2926/2547	0.93	0.67–1.29	0.6580	45.3%
All stroke/TIA	5	2926/2547	1.06	0.81–1.38	0.6876	16.2%
Ischemic stroke/TIA	4	1427/1046	1.24	0.88–1.76	0.2191	0.0%
Systemic embolism	4	2194/2165	0.76	0.12–4.77	0.7729	16.0%
Major bleeding	6	3729/3344	0.93	0.77–1.13	0.4958	8.5%
Major bleeding not related to device/procedure	4	3235/2881	0.54	0.46–0.63	<0.0001	0.0%

CI, confidence interval; RR, risk ratio; TIA, transient ischemic attack.

**Table 4:** Descriptive procedural and device-related complications by included trial.

Study	Implantation Attempted/Successful, n (%)	Periprocedural Stroke/TIA	Pericardial Effusion/Tamponade	Vascular Complications	Device Embolization	Device-Related Thrombus	Peridevice Leak	Procedure-Related Bleeding	Death (≤7 d)
CLOSURE-AF (Landmesser 2026) [18]	421/446 attempted; 406/421 successful (96.4%)	1 procedure-related TIA	5 (4 pericardiocentesis, 1 surgical)	1 (peripheral embolism)	1 (surgical removal)	NR	20 leaks by day 7 (16 < 3 mm, 1 3–5 mm, 3 >5 mm)	18 (BARC 3–5, transfusion)	2
CHAMPION-AF (Doshi 2026) [17]	1499 assigned; 1386 received device (92.5%); 22 unsuccessful	NR ^1^	10 effusions requiring intervention (0.7%), ≤30 d	NR	NR	63/1320 imaged (4.8%); 24 (1.8%) clinically relevant; 2 strokes	14/1012 (1.4%) > 3 mm at 4 mo	NR ^1^	NR ^1^
OPTION (Wazni 2025) [16]	762 attempted; 753 successful (98.8%)	NR ^2^	~0.3% (2 pts)	NR	NR	1.9% at 12 mo	~19–21% any leak (complete seal 81.0% → 79.7%)	NR ^2^	NR ^2^
PRAGUE-17 (Osmancik 2020) [15]	187 attempted; 181 successful (96.8%)	0 intraprocedural	2 (both late: 89 d, 194 d)	2 (1 pseudoaneurysm, 1 groin hematoma)	1 (acute, surgical)	6/178 imaged at 3 mo (3.4%)	4 (2.2%) >5 mm; 20 (11.2%) 1–5 mm at 3 mo	Included under vascular/death	1 procedure-related; 1 device-related (late tamponade, ~6 wk)
COMPARE-LAAO (Aarnink 2026) [19]	40 attempted; 39 successful (97.5%)	1 (device migration → ischemic stroke) ^3^	1 (pericardiocentesis)	0 separately listed	1 (percutaneous retrieval) ^3^	0 observed	8/37 (22%) at 3 mo; 7 <3 mm, 1 >5 mm	0	0
PROTECT AF (Holmes 2009) [12] ^4^	449 attempted; 408 successful (91%)	5 (1.1%) procedure-related ischemic stroke	22 (4.8%) serious effusions (15 pericardiocentesis, 7 surgical)	NR; 2 other (0.4%; esophageal tear, arrhythmia)	3 (0.6%; 1 percutaneous, 2 surgical)	NR	NR as discrete outcome	16 (3.5%) major bleeding	0 attributed to device
PREVAIL (Holmes 2014) [13] ^4^	265 attempted; 252 successful (95.1%)	NR (narrow endpoint); 0.7% per cross-trial Comparison ^5^	1 (0.4%) tamponade; cross-trial comparison: 0.4% surgical + 1.5% pericardiocentesis	1 (0.4%) AV fistula; 1 (0.4%) cardiac perforation	2 (0.7%)	NR	NR	1 (0.4%) major bleed, transfusion	0 among primary safety-endpoint events

NR, not reported in the source publication’s main text (may be reported only in a supplementary appendix not available for extraction); BARC, Bleeding Academic Research Consortium; AV, arteriovenous. ^1^ Reported in supplementary appendix only (Table S20), not extractable from main text. ^2^ Itemized procedural complications not given in main text (Table S12). ^3^ Both events occurred in the same patient (device migration shortly after implantation, percutaneously retrieved, resulting in ischemic stroke). ^4^ Reddy 2017 [14], the pooled patient-level meta-analysis of PROTECT AF and PREVAIL included in this review, reports pooled long-term efficacy and safety outcomes but no new procedural-complication breakdown; rows shown are therefore sourced directly from the original PROTECT AF [12] and PREVAIL [13] trial publications. ^5^ The PREVAIL publication’s abstract states a composite 7-day procedural complication rate of 4.2% versus PROTECT AF’s 8.7%; however, Table 7 of the same publication lists PREVAIL’s own rate as 4.5%, with 4.2% corresponding to the CAP registry (a third, non-randomized comparator arm). The trial-specific value of 4.5% is used here.

**Table 5:** GRADE summary of findings for the main clinical outcomes.

Outcome (Studies/Participants)	Risk of Bias	Inconsistency	Indirectness	Imprecision	Publication Bias	Certainty (GRADE)	Relative Effect (95% CI)
Composite primary endpoint (6 studies, n = 7073)	Serious ^1^	Not serious (I^2^ = 32.0%)	Serious ^2^	Not serious	Undetected ^3^	Low	RR 1.02 (0.85–1.23)
All-cause death (5 studies, n = 6671)	Serious ^1^	Serious (I^2^ = 42.3%)	Serious ^2^	Not serious	Undetected ^3^	Very low	RR 1.02 (0.79–1.31)
Cardiovascular death (5 studies, n = 5473)	Serious ^1^	Serious (I^2^ = 45.3%)	Serious ^2^	Serious ^4^	Undetected ^3^	Very low	RR 0.93 (0.67–1.29)
All stroke/TIA (5 studies, n = 5473)	Serious ^1^	Not serious (I^2^ = 16.2%)	Serious ^2^	Not serious	Undetected ^3^	Low	RR 1.06 (0.81–1.38)
Ischemic stroke/TIA (4 studies, n = 2473)	Serious ^1^	Not serious (I^2^ = 0.0%)	Serious ^2^	Serious ^5^	Undetected ^3^	Very low	RR 1.24 (0.88–1.76)
Systemic embolism (4 studies, n = 4359)	Serious ^1^	Not serious (I^2^ = 16.0%)	Serious ^2^	Very serious ^6^	Undetected ^3^	Very low	RR 0.76 (0.12–4.77)
Major bleeding, total (6 studies, n = 7073)	Serious ^1^	Not serious (I^2^ = 8.5%)	Serious ^2^	Not serious	Undetected ^3^	Low	RR 0.93 (0.77–1.13)
Nonprocedural major bleeding (4 studies, n = 6116)	Serious ^1^	Not serious (I^2^ = 0.0%)	Serious ^2^	Not serious	Undetected ^3^	Low	RR 0.54 (0.46–0.63)

CI, confidence interval; GRADE, Grading of Recommendations Assessment, Development and Evaluation; RR, risk ratio; TIA, transient ischemic attack. ^1^ Downgraded for risk of bias: four of six included trials were rated as having some concerns or high risk of bias on ROB 2 (Section 3.3), and an open-label design is inherent to all LAAO-versus-medical-therapy comparisons. ^2^ Downgraded for indirectness: pooled trials compared LAAO against heterogeneous medical-therapy regimens (warfarin, direct oral anticoagulants, antiplatelet therapy, or no antithrombotic therapy) in populations differing in anticoagulation eligibility and baseline risk. ^3^ Not downgraded for publication bias; formal testing was judged to have limited interpretive value given six or fewer studies per outcome (Section 3.9). ^4^ Downgraded for imprecision: confidence interval spans both a clinically important reduction and a clinically important increase in cardiovascular death. ^5^ Downgraded for imprecision: confidence interval is compatible with a clinically meaningful near-doubling of ischemic stroke/TIA risk with LAAO. ^6^ Downgraded twice (very serious imprecision): very wide confidence interval reflecting few events, compatible with both substantial benefit and substantial harm. These ratings reflect a conservative, team-reviewable judgment; risk-of-bias and indirectness downgrades in particular should be confirmed by the study team against the per-outcome meta-analytic weights.

## Data Availability

No new data was created in this study. All data analyzed in this meta-analysis were extracted from the previously published randomized controlled trials cited in the reference list [12,13,14,15,16,17,18,19]. The extracted data supporting the findings of this study are available within the article and its Supplementary Materials, or from the corresponding author upon reasonable request.

## Supplementary Materials

The following supporting information can be downloaded at: https://www.mdpi.com/article/10.3390/jcm15145529/s1. Supplementary Table S1. PRISMA 2020 Checklist. Table S2: Full electronic search strategy. Figure S1: PRISMA 2020 study selection flow diagram. Figure S2: Risk-of-bias (ROB 2) summary assessment. Figure S3: Forest plot for the composite primary endpoint. Figure S4: Forest plot for all-cause death. Figure S5: Forest plot for cardiovascular death. Figure S6: Forest plot for all stroke/TIA. Figure S7: Forest plot for ischemic stroke/TIA. Figure S8: Forest plot for systemic embolism. Figure S9: Forest plot for major bleeding.
