# Supplementary material for: Left Atrial Appendage Occlusion Versus Medical Therapy in Atrial Fibrillation: A Systematic Review and Meta-Analysis
Source: J Clin Med. 2026 Jul 15;15(14):5529. doi: 10.3390/jcm15145529 (PMC13413204; doi:10.3390/jcm15145529)

Left Atrial Appendage Occlusion Versus Medical Therapy in Atrial Fibrillation: A Systematic Review and Meta-Analysis

Muhammad Aslam Khan MD; Anza Muhammad MBBS; Sheeza Nawaz MD; Muhammad Khan Buhadur Ali MBBS; Muhammad Shahzaib MBBS; Aleena Sameen MBBS; Maheen Anwar MD; Akriti Agarwal MD; Syed Zamrak Khan MD; Saurabh Sharma MD

Supplementary Table S1. PRISMA 2020 Checklist

Section and Topic	Item #	Checklist Item	Location in Manuscript
**TITLE**			
Title	**1**	Identify the report as a systematic review.	Title page — manuscript title explicitly states 'Systematic Review and Meta-Analysis'
**ABSTRACT**			
Abstract	**2**	See the PRISMA 2020 for Abstracts checklist.	Abstract — structured with Background, Methods, Results, Conclusions; PROSPERO number included
**INTRODUCTION**			

Rationale	**3**	Describe the rationale for the review in the context of existing knowledge.	Section 1 (Introduction), paragraphs 1–3
Objectives	**4**	Provide an explicit statement of the objective(s) or question(s) the review addresses.	Section 1 (Introduction), paragraph 3 — hypothesis and objective stated explicitly
**METHODS**			
Eligibility criteria	**5**	Specify the inclusion and exclusion criteria for the review and how studies were grouped for the syntheses.	Section 2.3 (Eligibility Criteria) — inclusion and exclusion criteria listed; all eligible studies pooled in a single synthesis comparing LAAO vs medical therapy
Informatio n sources	**6**	Specify all databases, registers, websites, organisations, reference lists and other sources searched or consulted to identify studies. Specify the date when each source was last searched or consulted.	Section 2.2 (Search Strategy) — PubMed, CENTRAL, ScienceDirect; searched from inception through May 2026

Search strategy	**7**	Present the full search strategies for all databases, registers and websites, including any filters and limits used.	Supplementary Table S1 — full search strings for PubMed, Cochrane CENTRAL, and ScienceDirect
Selection process	**8**	Specify the methods used to decide whether a study met the inclusion criteria of the review, including how many reviewers screened each record and each report retrieved, whether they worked independently, and if applicable, details of automation tools used in the process.	Section 2.4 (Study Selection and Data Extraction) — two independent reviewers (AM and AS) screened titles, abstracts, and full texts; disagreements resolved by consensus; no automation tools used
Data collection process	**9**	Specify the methods used to collect data from reports, including how many reviewers collected data from each report, whether they worked independently, any processes for obtaining or confirming data from study investigators, and if applicable, details of automation tools used in the process.	Section 2.4 (Study Selection and Data Extraction) — two reviewers collected data using a standardized extraction form; no automation tools used

Data items — outcomes	**10a**	List and define all outcomes for which data were sought. Specify whether all results that were compatible with each outcome domain in each study were sought (e.g. for all measures, time points, analyses), and if not, the methods used to decide which results to collect.	Section 2.5 (Outcomes) — all prespecified outcomes listed; event counts and denominators extracted as reported by each trial
Data items — other variables	**10b**	List and define all other variables for which data were sought (e.g. participant and intervention characteristics, funding sources). Describe any assumptions made about any missing or unclear information.	Section 2.4 (Study Selection and Data Extraction) and Tables 2A–2B — participant characteristics, intervention/comparator, sample size, sex, age, BMI, CHA2DS2-VASc, AF pattern, comorbidities; N/A used where data not reported by trial

Study risk of bias assessment	**11**	Specify the methods used to assess risk of bias in the included studies, including details of the tool(s) used, how many reviewers assessed each study and whether they worked independently, and if applicable, details of automation tools used in the process.	Section 2.6 (Risk of Bias Assessment) — ROB 2 tool used; two reviewers assessed each study independently; domains rated as low risk, some concerns, or high risk
Effect measures	**12**	Specify for each outcome the effect measure(s) (e.g. risk ratio, mean difference) used in the synthesis or presentation of results.	Section 2.7 (Statistical Analysis) — risk ratio (RR) with 95% CI used for all dichotomous outcomes
Synthesis methods — eligibility for synthesis	**13a**	Describe the processes used to decide which studies were eligible for each synthesis (e.g. tabulating the study intervention characteristics and comparing against the planned groups for each synthesis (item #5)).	Section 2.3 and 2.7 — all RCTs meeting eligibility criteria and reporting the relevant outcome were included in each synthesis; intervention and comparator characteristics tabulated in Table 1

Synthesis methods — data preparation	**13b**	Describe any methods required to prepare the data for presentation or synthesis, such as handling of missing summary statistics, or data conversions.	Section 2.7 (Statistical Analysis) — binary event counts and denominators extracted directly; no data conversions required; missing data indicated as N/A in tables
Synthesis methods — tabulating/ displaying results	**13c**	Describe any methods used to tabulate or visually display results of individual studies and syntheses.	Table 3 (pooled estimates); Supplementary Figures S3–S9 (forest plots for each outcome); Tables 2A–2B (individual study characteristics)
Synthesis methods — synthesis model	**13d**	Describe any methods used to synthesize results and provide a rationale for the choice(s). If meta-analysis was performed, describe the model(s), method(s) to identify the presence and extent of statistical heterogeneity, and software package(s) used.	Section 2.7 (Statistical Analysis) — DerSimonian-Laird random-effects model; heterogeneity assessed using I^2, τ^2, and Cochran Q test; R version 4.5.0 with packages meta and rtsa [28,29]

Synthesis methods — heterogeneity exploration	**13e**	Describe any methods used to explore possible causes of heterogeneity among study results (e.g. subgroup analysis, meta-regression).	Section 2.7 and Limitations — formal subgroup analyses and meta-regression were not performed due to the limited number of included trials and use of trial-level aggregate data; sources of heterogeneity discussed narratively in Section 4 (Discussion)
Synthesis methods — sensitivity analyses	**13f**	Describe any sensitivity analyses conducted to assess robustness of the synthesized results.	Section 2.7 (Statistical Analysis) — leave-one-out sensitivity analyses performed for each outcome; results reported in Section 3.8 and Supplementary Figures B1–B8

Reporting bias assessment	**14**	Describe any methods used to assess risk of bias due to missing results in a synthesis (arising from reporting biases).	Section 2.7 (Statistical Analysis) — funnel plots generated for all eight outcomes; Supplementary Figures A1–A8; formal Egger test not performed due to insufficient number of studies per outcome [26]
Certainty assessment	**15**	Describe any methods used to assess certainty (or confidence) in the body of evidence for an outcome.	Section 2.7 (Statistical Analysis) — formal certainty of evidence assessment using the GRADE approach was not performed as this was beyond the scope of the current analysis
**RESULTS**			

Study selection — results	**16a**	Describe the results of the search and selection process, from the number of records identified in the search to the number of studies included in the review, ideally using a flow diagram.	Section 3.1 (Study Selection) — 1,809 records identified; 596 duplicates removed; 6 RCTs included; PRISMA flow diagram in Supplementary Figure S1
Study selection — excluded studies	**16b**	Cite studies that might appear to meet the inclusion criteria, but which were excluded, and explain why they were excluded.	Section 3.1 (Study Selection) — studies excluded after full-text review were primarily non-randomized in design, reported duplicate data from included trials, or did not report any prespecified clinical outcome; full list available from corresponding author upon request

Study characteristics	**17**	Cite each included study and present its characteristics.	Section 3.2 (Study Characteristics) and Table 1 — all six included studies cited with trial name, intervention, and comparator; Tables 2A–2B present baseline characteristics
Risk of bias in studies	**18**	Present assessments of risk of bias for each included study.	Section 3.3 (Risk of Bias) and Supplementary Figure S2 — ROB 2 judgements for all six included studies presented individually
Results of individual studies	**19**	For all outcomes, present, for each study: (a) summary statistics for each group (where appropriate) and (b) an effect estimate and its precision (e.g. confidence/credible interval), ideally using structured tables or plots.	Supplementary Figures S3–S9 (forest plots showing individual study estimates and pooled effects for each outcome); Table 3 (pooled estimates)

Results of syntheses — characteristics summary	**20a**	For each synthesis, briefly summarise the characteristics and risk of bias among contributing studies.	Sections 3.2, 3.3, and 3.4 — contributing study characteristics and ROB 2 judgements summarized for each outcome synthesis
Results of syntheses — statistical results	**20b**	Present results of all statistical syntheses conducted. If meta-analysis was done, present for each the summary estimate and its precision (e.g. confidence/credible interval) and measures of statistical heterogeneity. If comparing groups, describe the direction of the effect.	Table 3 and Sections 3.4–3.7 — RR, 95% CI, p value, I^2, and τ^2 reported for all eight outcomes
Results of syntheses — heterogeneity causes	**20c**	Present results of all investigations of possible causes of heterogeneity among study results.	Section 4 (Discussion), heterogeneity paragraph — sources of heterogeneity for mortality outcomes discussed narratively (warfarin-era vs DOAC-era comparisons, baseline risk differences, procedural eras); formal meta-regression not performed

Results of syntheses — sensitivity analyses	**20d**	Present results of all sensitivity analyses conducted to assess the robustness of the synthesized results.	Section 3.8 (Sensitivity Analyses) and Supplementary Figures B1–B8 — leave-one-out RR ranges reported for all outcomes
Reporting biases	**21**	Present assessments of risk of bias due to missing results (arising from reporting biases) for each synthesis assessed.	Section 3.9 (Publication Bias) and Supplementary Figures A1–A8 — funnel plots generated and assessed for all eight outcomes; formal quantitative testing limited by small number of studies
Certainty of evidence	**22**	Present assessments of certainty (or confidence) in the body of evidence for each outcome assessed.	Limitations section — GRADE certainty assessment was not performed as this was beyond the scope of the current analysis
**DISCUSSION**			

Discussion — interpretation	**23a**	Provide a general interpretation of the results in the context of other evidence.	Section 4 (Discussion), paragraphs 1–5 — results interpreted in context of CHAMPION-AF, CLOSURE-AF, PRAGUE-17, OPTION, PREVAIL/PROTECT AF, and prior meta-analyses
Discussion — limitations of evidence	**23b**	Discuss any limitations of the evidence included in the review.	Limitations subsection — six limitations discussed including trial-level data, small number of trials, different follow-up durations, and heterogeneous populations

Discussion — limitations of review processes	**23c**	Discuss any limitations of the review processes used.	Limitations subsection — use of trial-level aggregate data precluding individual patient-level subgroup analyses; small number of studies limiting formal publication bias assessment
Discussion — implications	**23d**	Discuss implications of the results for practice, policy, and future research.	Section 5 (Conclusions) — individualized treatment selection recommended; future extended follow-up studies identified
**OTHER INFORMATION**			
Registration and protocol — registration	**24a**	Provide registration information for the review, including register name and registration number, or state that the review was not registered.	Abstract (final line) and Section 2.1 — PROSPERO registration number CRD420261403066

Registration and protocol — protocol access	**24b**	Indicate where the review protocol can be accessed, or state that a protocol was not prepared.	Section 2.1 (Reporting Framework) — protocol accessible via PROSPERO database (CRD420261403066)
Registration and protocol — amendments	**24c**	Describe and explain any amendments to information provided at registration or in the protocol.	Section 2.1 (Reporting Framework) — no amendments were made to the registered protocol
Support	**25**	Describe sources of financial or non-financial support for the review, and the role of the funders or sponsors in the review.	Funding statement — this research did not receive any specific grant from funding agencies in the public, commercial, or not-for-profit sectors
Competing interests	**26**	Declare any competing interests of review authors.	Declaration of Competing Interests — all authors declare no competing interests

Availability of data, code and other materials	**27**	Report which of the following are publicly available and where they can be found: template data collection forms; data extracted from included studies; data used for all analyses; analytic code; any other materials used in the review.	Data Availability Statement — all data derived from previously published RCTs available in original publications cited in the reference list; analytic code available from corresponding author upon request

From: Page MJ, McKenzie JE, Bossuyt PM, et al. The PRISMA 2020 statement: an updated guideline for reporting systematic reviews. BMJ 2021;372:n71. doi:10.1136/bmj.n71

Supplementary Table S2. Database search strings.

PubMed:

(("Left Atrial Appendage Closure"[Mesh] OR "LAA closure"[tiab] OR "left atrial appendage occlusion"[tiab] OR Watchman[tiab] OR LARIAT[tiab] OR Amplatzer[tiab] OR LAmbre[tiab]) AND ("Atrial Fibrillation"[Mesh] OR "atrial fibrillation"[tiab]) AND ("anticoagulation"[tiab] OR warfarin[tiab] OR dabigatran[tiab] OR apixaban[tiab] OR rivaroxaban[tiab] OR DOAC[tiab] OR antiplatelet*[tiab] OR aspirin[tiab]))

Cochrane (CENTRAL):

Step	Query
#1	[mh "Left Atrial Appendage Closure"]
#2	("LAA closure" OR "left atrial appendage occlusion" OR Watchman OR LARIAT OR Amplatzer OR LAmbre):ti,ab,kw
#3	#1 OR #2
#4	[mh "Atrial Fibrillation"]
#5	("atrial fibrillation"):ti,ab,kw
#6	#4 OR #5
#7	("anticoagulation" OR warfarin OR dabigatran OR apixaban OR rivaroxaban OR DOAC OR antiplatelet* OR aspirin):ti,ab,kw
#8	#3 AND #6 AND #7

ScienceDirect:

("appendage occlusion" OR LARIAT OR LAmbre) AND "atrial fibrillation" AND (anticoagulation OR DOAC OR aspirin)

Supplementary Figure S1. PRISMA 2020 flow diagram for study selection.

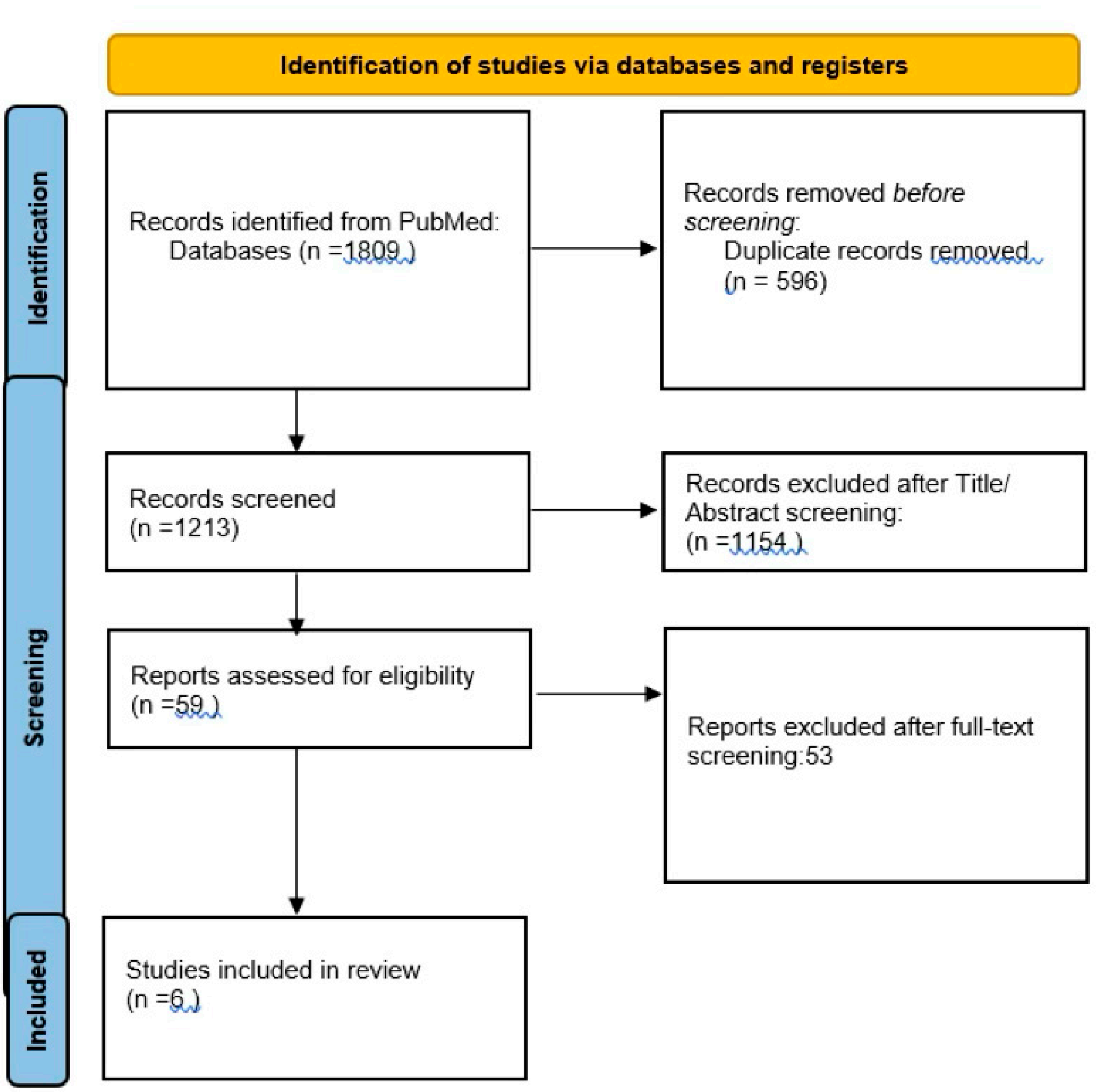

Supplementary Figure S2. Traffic-light plot of ROB 2 domain-level and overall risk-of-bias judgements for included studies.

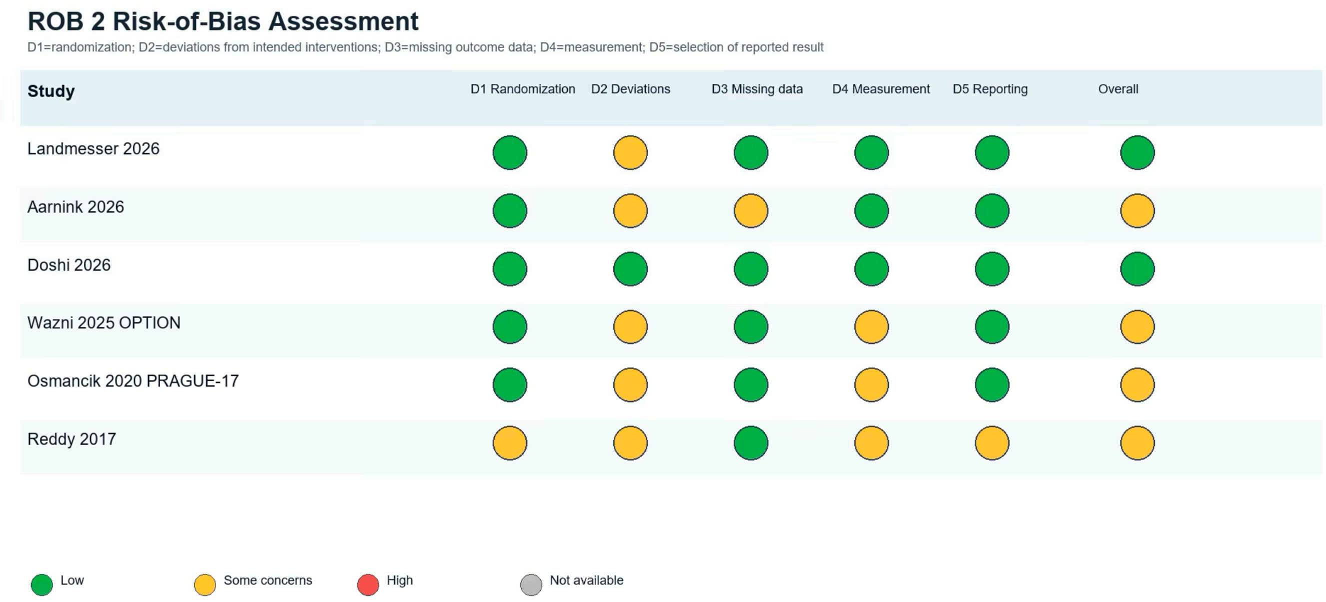

Supplementary Figure S3. Forest plot for the composite primary endpoint.

Study	LAAO Events	LAAO Total	Medical therapy Events	Medical therapy Total	RR	95%-CI	Weight
Landmesser 2026	155	446	127	442	1.21	[1.00; 1.47]	30.0%
Doshi 2026	81	1499	65	1501	1.25	[0.91; 1.72]	19.3%
Aarnink 2026	10	48	4	21	1.09	[0.39; 3.09]	3.0%
Wazni 2025	41	803	44	797	0.92	[0.61; 1.40]	13.8%
Osmanick 2020	38	201	47	201	0.81	[0.55; 1.18]	15.5%
Reddy 2017	79	732	50	382	0.82	[0.59; 1.15]	18.4%
**Random effects model**		**3729**		**3344**	**1.02**	**[0.85; 1.23]**	**100.0%**

Heterogeneity: $I^2 = 32.0\%$, $\tau^2 = 0.0199$, $p = 0.1960$
Test for overall effect: $z = 0.25$ ($p = 0.8056$)

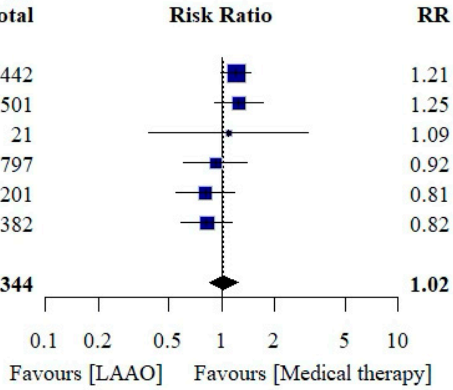

Supplementary Figure S4. Forest plot for all-cause death.

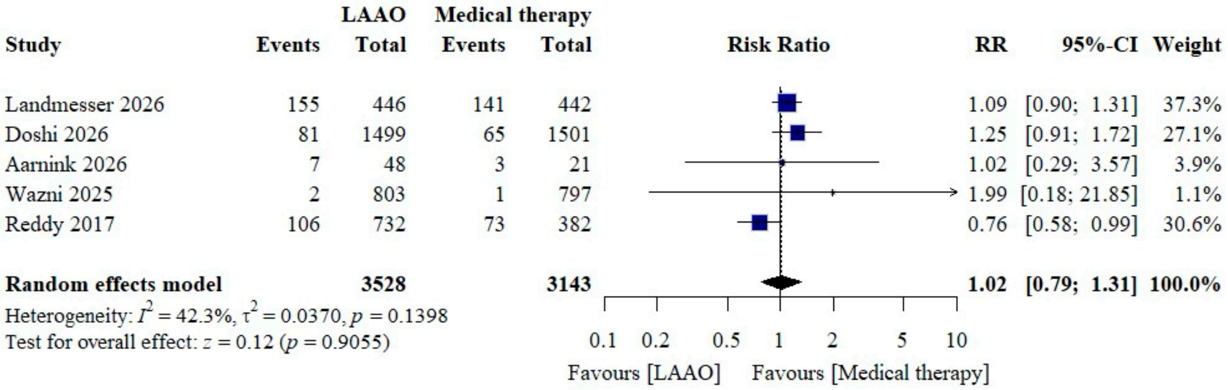

Supplementary Figure S5. Forest plot for cardiovascular death.

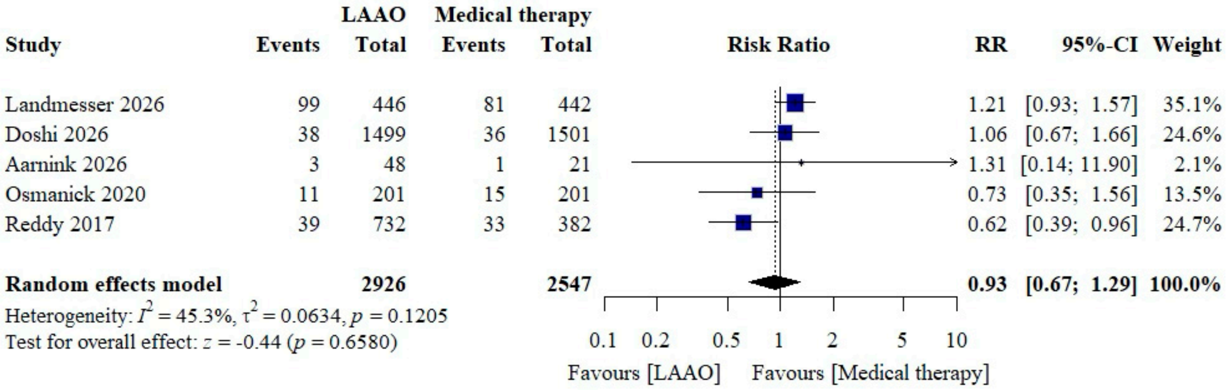

Supplementary Figure S6. Forest plot for all stroke/TIA.

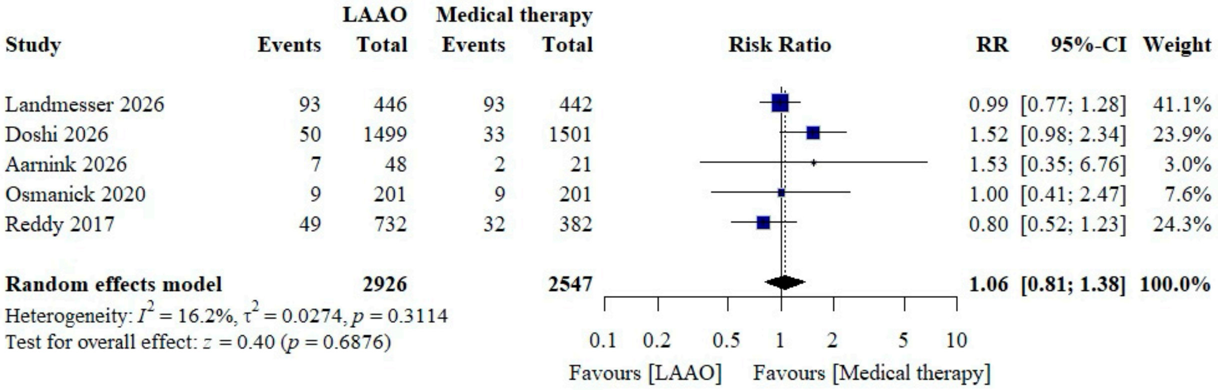

Supplementary Figure S7. Forest plot for ischemic stroke/TIA.

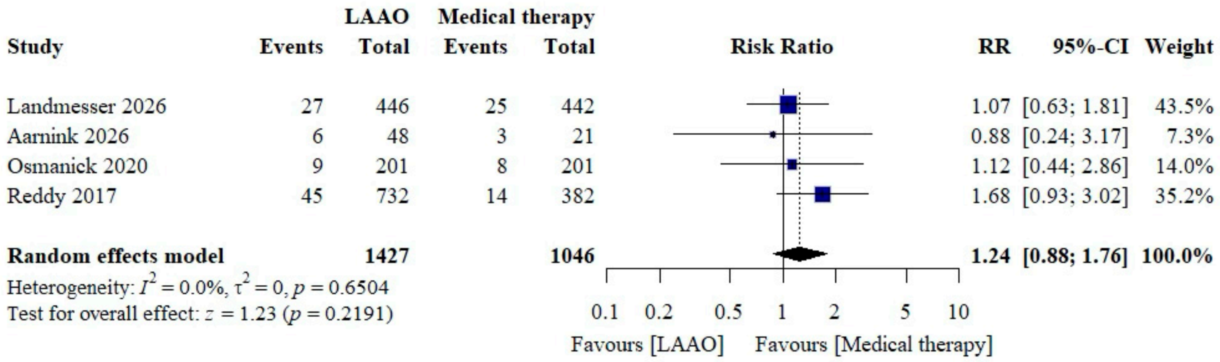

Supplementary Figure S8. Forest plot for systemic embolism.

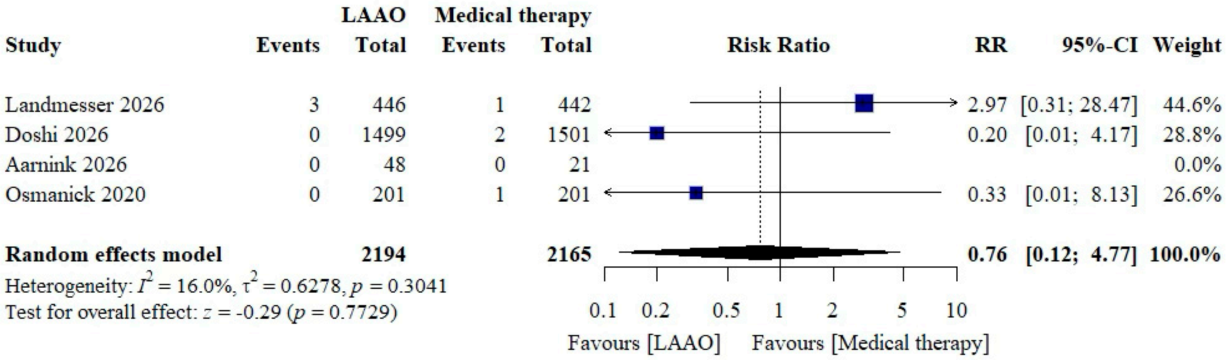

Supplementary Figure S9. Forest plot for major bleeding.

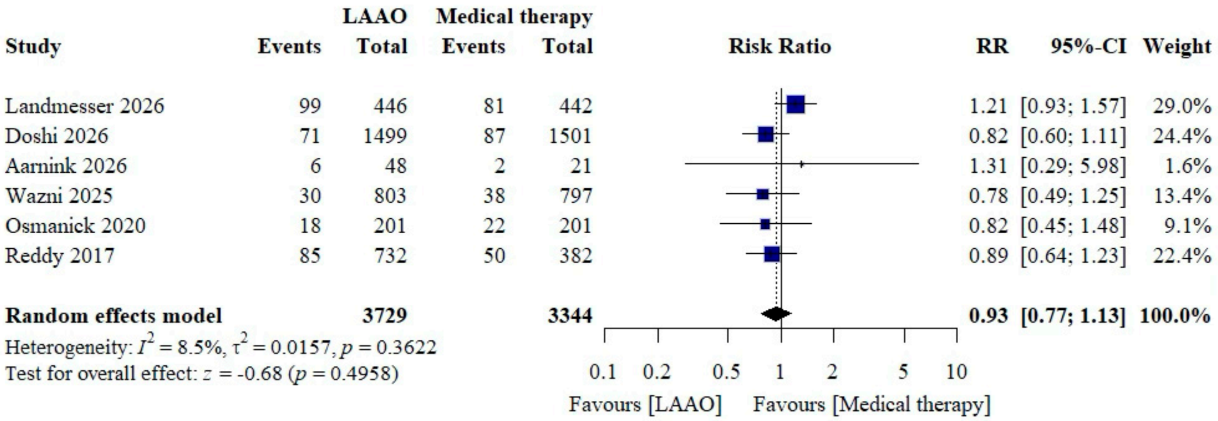

Supplement: Supplementary file 1 [file jcm-15-05529-s001.zip › jcm-4407791-supplementary.pdf]
